# Thrive With Me: Protocol for a Randomized Controlled Trial to Test a Peer Support Intervention to Improve Antiretroviral Therapy Adherence Among Men Who Have Sex With Men

**DOI:** 10.2196/10182

**Published:** 2018-05-31

**Authors:** Keith J Horvath, K Rivet Amico, Darin Erickson, Alexandra M Ecklund, Aldona Martinka, James DeWitt, Jeffery McLaughlin, Jeffrey T Parsons

**Affiliations:** ^1^ Division of Epidemiology and Community Health School of Public Health University of Minnesota Minneapolis, MN United States; ^2^ Department of Health Behavior and Health Education School of Public Health University of Michigan Ann Arbor, MI United States; ^3^ Division of Health Policy and Management School of Public Health University of Minnesota Minneapolis, MN United States; ^4^ Radiant Creative Group Houston, TX United States; ^5^ Center for HIV/AIDS Educational Studies and Training (CHEST) Hunter College Graduate Center of the City University of New York (CUNY) New York City, NY United States

**Keywords:** ART adherence, MSM, mobile app, HIV

## Abstract

**Background:**

The suboptimal rate of viral suppression among persons aged 13 years and older and residing in 37 states and the District of Columbia leaves considerable opportunities for onward transmission and contributes to poor health outcomes. Men who have sex with men (MSM) represent one of the most at-risk groups in the United States. There is a clear and continued need for innovative adherence support programs to optimize viral suppression. To address this gap, we designed and are implementing a randomized controlled trial (RCT) to test the efficacy of the Thrive with Me intervention for MSM living with HIV. Critical components of the protocol are presented.

**Objective:**

The aim of this study is to describe the protocol for rigorously testing the efficacy of Thrive with Me to improve antiretroviral therapy (ART) adherence among HIV-positive MSM residing in New York City.

**Methods:**

A community advisory board and beta testing were used to obtain feedback from HIV-positive MSM on the overall look and feel of Thrive with Me and problems with navigation to finalize intervention components and content. We will enroll 400 HIV-positive MSM residing in the New York City area into a two-arm prospective RCT and follow them for 17 months. Men in the Thrive with Me experimental intervention arm will have access to Thrive with Me for 5 months. Thrive with Me has three primary components: (1) a private social networking feature; (2) tailored HIV and ART adherence information; and (3) medication reminders, self-monitoring, and reflection. Gamification components include badges and leveling up to increase intrinsic motivation to engage with the intervention. Men randomized to the control condition will view a weekly newsletter for 5 months. The newsletter will be delivered via email and contains information on topics related to HIV with the exception of ART adherence. Study assessments will occur at enrollment and 5, 11, and 17 months post enrollment. The primary study outcome is HIV viral load, which is considered an objective indicator of ART adherence.

**Results:**

Participant recruitment for the RCT began in October 2016, and the data collection period is anticipated to end in the Fall of 2019.

**Conclusions:**

The efficacy trial of Thrive with Me will help to fill gaps in understanding about the utility of multicomponent, technology-based interventions to improve ART adherence among HIV-positive MSM. Of importance is the ability for the results of the Thrive with Me trial to inform best practices for conducting technology-based interventions that incorporate social media features.

**Trial Registration:**

ClinicalTrials.gov NCT02704208; https://clinicaltrials.gov/ct2/show/NCT02704208 (Archived by WebCite at http://www.webcitation.org/6zQ8WPra6)

**Registered Report identifier:**

RR1-10.2196/10182

## Introduction

### Background

HIV rates in the United States, for the most recent 5 years data that are available, have declined slightly from 14.2 new HIV diagnoses per 100,000 in 2010 to 12.3 in 2015 [1]. Despite decreases in HIV infection rates among injection drug users, women, and heterosexual men, those attributed to male-to-male sexual contact remain stable. In 2015, 70% of all new HIV infections were attributed to male-to-male sexual contact (including the category of male-to-male sexual contact and injection drug use) [1]. Current US Guidelines for the Use of Antiretroviral Agents [2] state that undetectable viral load (VL) “is one of the most reliable indicators of adherence,” which is largely achieved though sufficient and sustained adherence to antiretroviral therapy (ART). Optimal ART adherence reduces excess morbidity and mortality among people living with HIV or AIDS (PLWH) [3] and lowers the probability of forward transmission to sexual partners [4]. However, in a review of studies of the treatment cascade for men who have sex with men (MSM), viral suppression was between 16% and 42% among the 7 reported studies conducted in the United States [5]. The most recent Centers for Disease Control and Prevention (CDC) surveillance data showed that 61% of MSM diagnosed with HIV in 37 states and the District of Columbia had VL below 200 copies/mL [6], although viral suppression is estimated to be lower among all people aware and unaware of their HIV-positive status (49%) [7]. Despite the disproportionate burden of HIV in MSM communities, only three of 13 medication adherence interventions included in CDC’s Compendium of Evidence-based Behavioral Interventions include a majority MSM participants; the first is a provider implemented approach evaluated over a decade ago [8], the second is an intensive one-on-one counseling approach [9], and the third is an individual-level, computerized, clinic-based counseling intervention [10]. Thus, advancing tailored and innovative ART adherence interventions for HIV-positive MSM remains a high priority [11].

The use of technology has rapidly grown since 2000, when only 52% of US adults used the internet [12]. Currently, 88% of US adults are online [12], 95% own a cell phone [13], 75% own a smartphone [13], and 68% use Facebook [14]. Technology-based ART adherence approaches have proliferated in recent years [15,16] because of the widespread adoption of technology across sociodemographic groups [12], their ability to reach a broad audience, and their low implementation costs [17]. However, computerized ART adherence interventions [10,18], including those specifically for MSM [19] and illicit drug users [20], tend to be individually delivered and do not leverage peer-to-peer interactivity that has come to symbolize Web 2.0 [19]. Peer support interventions may facilitate user-generated content through wall or message board posts that create unique social incentives to maintain high engagement in the intervention.

In-person peer support ART adherence interventions have shown moderate success in improving adherence outcomes [21,22]. Simoni and colleagues randomized 224 HIV-positive patients at a public HIV specialty clinic in Seattle, Washington to receive either in-person peer support, pager messaging, both in-person peer support and pager messaging, or usual care [22] for a 3-month period. Assessments occurred every 3 months for a total of 9 months. Participants who received the peer intervention reported higher self-reported adherence at the immediate postintervention assessment, although intervention effects diminished at later assessment periods. Only the pager intervention showed significant effects for improving biological outcomes, suggesting that other adherence supports may be needed to bolster peer support–only interventions.

In response to the growing use of technology and the limitations of in-person ART adherence peer support interventions, we developed and pilot tested the Thrive with Me (TWM) intervention. TWM is a virtual (ie, online) behavioral intervention that includes peer-to-peer communication features, tailored ART and HIV information, and an ART self-monitoring component. TWM is grounded in the information- motivation-behavioral Skills (IMB) model [23,24]. We conducted a pilot study of TWM from February 2010 to April 2010 to assess its feasibility, acceptability, and preliminary efficacy among 123 adult MSM (mean age=43 years; 64.2%, 79/123 white) mostly recruited online in the United States. A full description of the study and its results has been published [25]. Briefly, results showed that 90.2% (111/123) were retained at the final assessment, and there were high acceptability ratings among men assigned to the TWM condition. Although overall adherence scores showed modest improvement from baseline to follow-up among the TWM intervention group compared with the control condition, these differences were not statistically significant. Among MSM who reported recent (<30 days) illicit drug use (16.3%, 20/123 of the sample), those in the TWM condition reported significantly higher overall ART adherence and ART taken correctly with food than those in the control condition.

**Figure 1:**
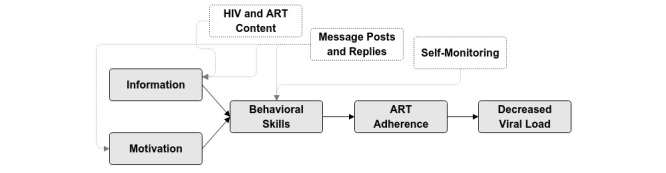
Thrive with Me intervention components and the information-motivation-behavioral skills (IMB) model. ART: antiretroviral therapy.

### Study Aims

The results of the TWM pilot study suggest that the approach of combining peer support, ART information, and adherence self-monitoring may improve ART adherence among MSM, especially those who use illicit drugs. This paper describes the protocol for a randomized controlled trial (RCT) to assess the efficacy of the TWM intervention to improve ART adherence among adult HIV-positive MSM. The aims of the study were as follows:

**Primary aim 1:** determine the efficacy of the TWM intervention to increase the proportion of virally suppressed HIV-positive MSM at postintervention time points.

*Hypothesis 1:* a higher proportion of participants in the TWM intervention than control participants will have undetectable VL at postintervention time points.

**Primary aim 2:** assess whether the TWM intervention is more beneficial for HIV-positive MSM who report recent drug use at baseline compared with HIV-positive MSM who do not.

*Hypothesis 2:* recent drug-using participants in the TWM intervention will demonstrate greatest improvements in VL and self-reported ART adherence at postintervention time points compared with nondrug-using participants.

We also propose the following secondary aims:

**Secondary aim 3:** examine the effects of the TWM intervention on sustained undetectable VL, defined as having an undetectable VL at all postbaseline assessment time points.

**Secondary aim 4:** examine the effects of the TWM intervention on theory-based change process (ie, IMB factors and social support) for improving VL, ART adherence, illicit drug use outcomes, and engagement in HIV care.

### Theoretical Basis for Intervention

The IMB model proposes that health behavior change occurs through the provision of relevant information, personal and social motivation to engage in the behavior, and appropriate behavioral skills to enact the behavior [26,27]. The associations between core TWM intervention components (described in detail below) and the IMB model components are shown in Figure 1.

The IMB model has been evaluated and supported using clinic-based samples in Puerto Rico [24], Italy [28], and Mississippi [29]. Using data collected as part of a pilot trial of TWM [30], our team investigated whether the IMB model is a useful predictive model of ART adherence among PLWH who were primarily recruited online and whether the theorized associations between IMB model constructs and adherence persisted in the presence of depression and current drug use [31]. Participants were on average 43 years of age, had been living with HIV for 9 or more years, and mostly male (84.0%, 270/312), white (68.8%, 222/312), and gay-identified (74.8%, 241/312). Using a revised version of the IMB scales, IMB constructs were associated with adherence as predicted by the theory among nondrug users and those with and without depression. However, among drug users, information exerted a direct effect on adherence but was not significantly associated with behavioral skills. These results suggest that the IMB model is an appropriate theoretical foundation for TWM, even when controlling for well-known determinants of adherence disruptions.

## Methods

### Design

**Figure 2:**
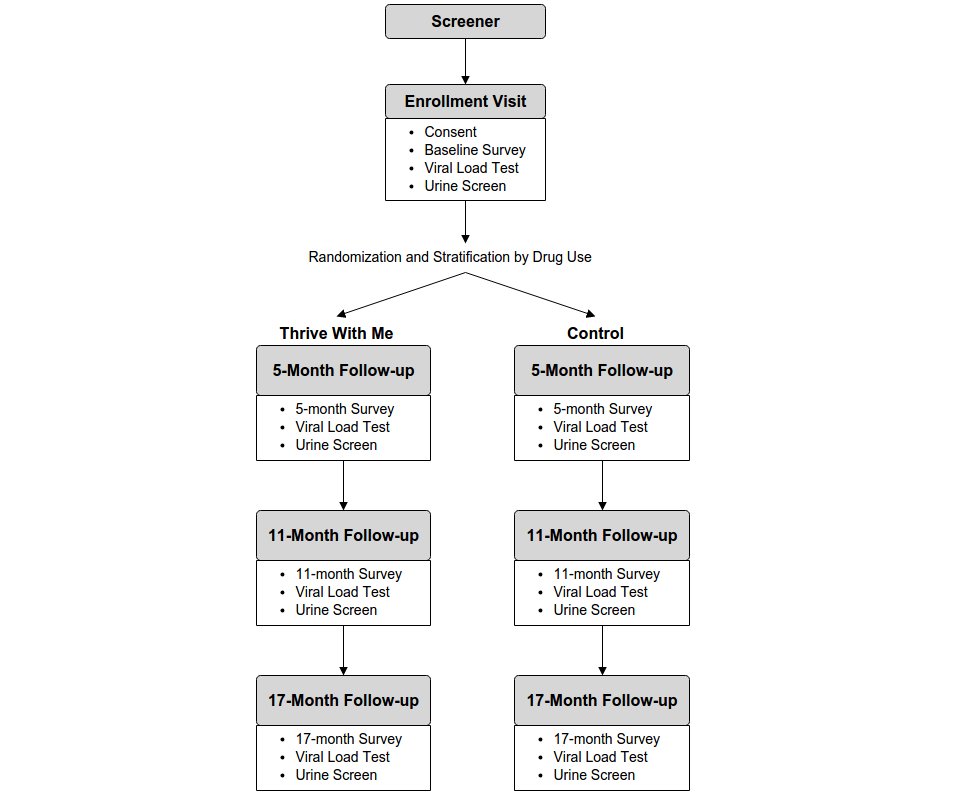
Participants' flow through the Thrive With Me study.

The research activities include a prospective two-arm RCT to test the efficacy of TWM to improve ART adherence among approximately 400 adult gay and bisexual MSM residing in the New York City area (Figure 2). TWM is a multisite study between the University of Minnesota (UMN) in Minneapolis and the Center for HIV Educational Studies and Training (CHEST), which is part of Hunter College, City University of New York. The team in Minnesota, led by principal investigator (PI) Dr Horvath, developed the TWM intervention, scientific protocols, and all training documents for implementation. The team at CHEST, led by coinvestigator Dr Parsons, manages the recruitment, enrollment, and retention of all participants. Following screening and enrollment, participants are randomized to either the experimental (TWM) or control arm. Men in the experimental arm receive access to the TWM intervention for a period of 5 months, whereas those in the control arm receive weekly emails with HIV-relevant content that does not include information about ART adherence. Assessments are conducted in-person at the CHEST offices by trained research personnel and occur for participants in both arms of the study at baseline, 5-month, 11-month, and 17-month follow-up time points. The primary outcome measure for the RCT is VL at the three follow-up assessment time points. We hypothesize that a higher proportion of participants in the TWM intervention than control participants will have undetectable VL at postintervention time points. We also hypothesize that recent drug-using participants in the TWM intervention will demonstrate greatest improvements in VL and self-reported ART adherence at postintervention time points compared with nondrug-using participants.

As secondary analyses, we will assess whether sustained undetectable VL, defined as having an undetectable VL across all follow-up assessment points, is higher among men in the TWM intervention condition. We will also assess the degree to which self-reported, theoretically derived adherence barriers (information, motivation, and behavioral skills) and social support within and outside the TWM intervention are associated with VL, self-reported ART adherence, illicit drug use outcomes, and engagement in HIV care.

### Participants

Men residing in the New York City metropolitan area will be recruited by CHEST staff to participate in this study. New York City was chosen as an ideal location to conduct the study because of the high numbers of HIV-infected MSM residents [1] and since only 38% of residents with HIV are estimated to be virally suppressed [32]. The eligibility criteria are shown in Textbox 1.

Additionally, a 50% target enrollment of drug-using (including powder cocaine, crack cocaine, painkillers, methamphetamine, heroin, hallucinogens, prescription drugs used recreationally, ketamine, MDMA, and poppers) men was established to ensure that both drug-using and nondrug-using men were enrolled in the trial. Due to its common use among HIV-positive and HIV-negative or unknown MSM [33], we did not include users who reported only marijuana use in the drug-using category.

**Eligibility criteria.**

Eligibility criteria

- Current male gender
- Having sex with a man in the past year
- HIV-positive serostatus (confirmed by current antiretroviral therapy prescription when possible)
- Reporting a detectable viral load (past year) or potentially suboptimal ART adherence (<90% in the past 30 days)
- English proficiency (as the intervention is in English)
- Residing in the New York City metropolitan area (so that men can attend in-person study visits)
- The ability to send and receive short message service messages
- Having internet access over the active (ie, 5 month) study period

### Randomization

Randomization occurs once participants are consented and enrolled in TWM. Participants complete a baseline computer-assisted survey instrument (CASI) programmed using Qualtrics (Provo, UT). At the end of the survey, a research assistant enters a password that enables Qualtrics to randomize the participant to either the experimental or control arm. Randomization was programmed using blocks of 20 (10 intervention and 10 control) to ensure we had enough users in the social network throughout data collection. After randomization, participants are further stratified by recent drug use and nonrecent drug use. Participants are not informed about their assigned arm in terms of intervention or active control, as the arms are described to participants simply as group 1 and group 2.

**Recruitment**

Participants will be recruited by staff at CHEST, which utilizes both active (eg, direct outreach) and passive (eg, advertising in clinic settings and internet-based) recruitment approaches to effectively recruit HIV+ MSM into intervention trials. CHEST has successfully developed a carefully monitored and organized recruitment structure through both internet- and field-based recruitment efforts. Recruitment efforts involve a preliminary screening process that significantly reduces the number of study-specific screeners conducted by project staff. CHEST uses internet recruitment efforts through online venues that cater to MSM and other populations that are most affected by HIV (eg, Grindr, Scruff, Adam4Adam, Craigslist, Facebook, Instagram, Twitter, and other social media platforms), as well as utilizing email distribution lists and blogs that expand to hard-to-reach populations through connections with nightlife party promoters, sex party promoters, and other community-based leaders or organizations. In addition to internet-based recruitment efforts, CHEST has utilized field-based recruitment strategies in their efforts for reaching HIV-positive MSM from diverse communities, ethnic and racial backgrounds, and sexual identity. Recruiters will conduct on-site preliminary screening via tablets at local bars, clubs, and community events frequented by gay and bisexual men in the New York City area. If a participant is determined to be preliminarily eligible for TWM, they are asked to provide contact information (including name, phone number, and email address) so that a study staff can contact them to perform the full study screener.

During the screening process, participants will be asked if they participated in the beta testing phase of TWM or are currently enrolled in TWM. Responding “yes” to either question will result in ineligibility. Eligible participants’ contact information (email, phone number, first and last name) will be automatically cross-referenced with enrolled participant information to capture duplicate participants before baseline and enrollment.

### Intervention

#### Thrive With Me Condition

Several strategies were employed to ensure sustainability, accessibility, and high user acceptance of TWM. To promote sustainability, the software team developed the program on a well-supported, open source, Web development framework and content management system, future-proofing the site against vendor lock-in and providing a platform for delivering a rich feature set within the resource constraints of the project. To promote accessibility, the user experience design team utilized a “responsive Web” approach, wherein the site layout dynamically adapts to suit the size and capability of the user’s device (eg, cell phone, tablet, or laptop). This ensured that TWM was available across a variety of devices and eliminated the need for multiple mobile device–specific codebases (eg, native apps). Finally, throughout development, the team sought input from representative users regarding the program interface, features, and design style, increasing the likelihood of high user acceptance upon release.

**Figure 3:**
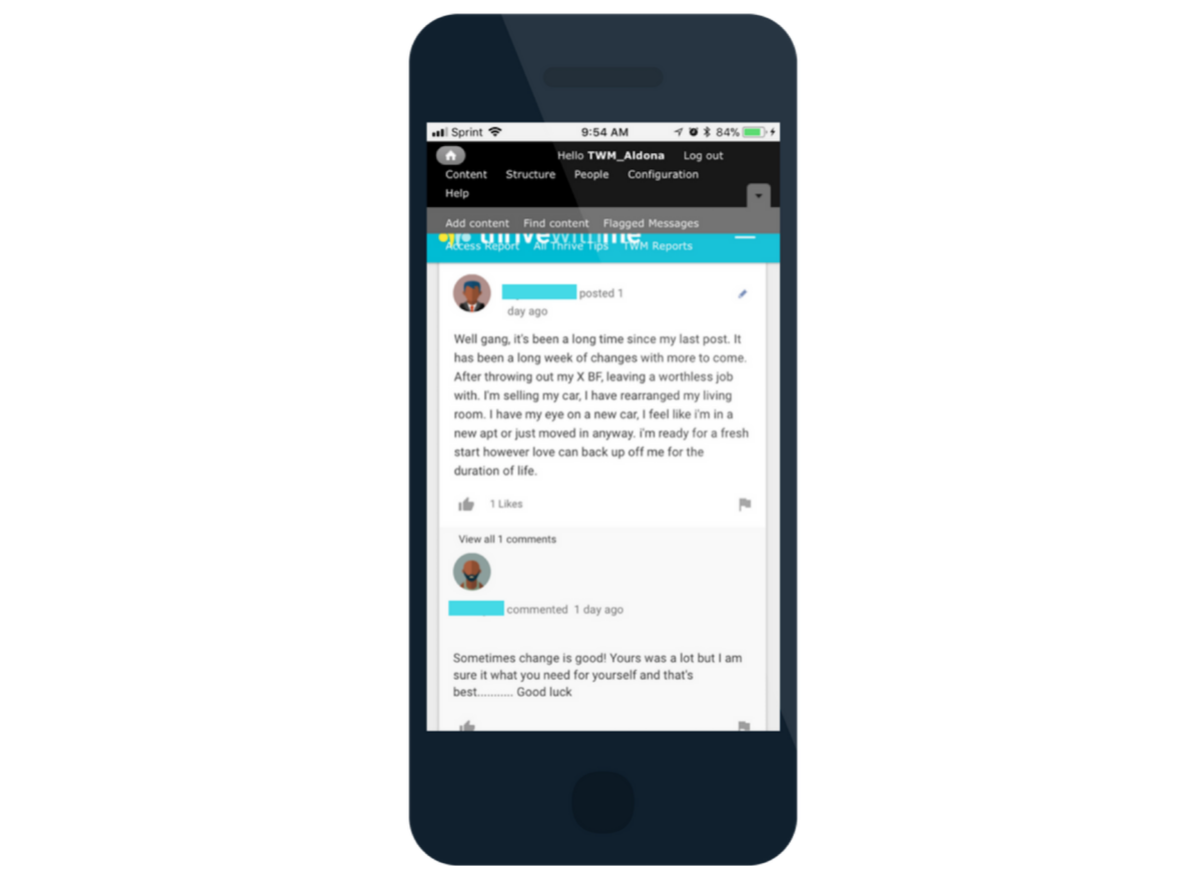
Thrive With Me peer interaction.

**Figure 4:**
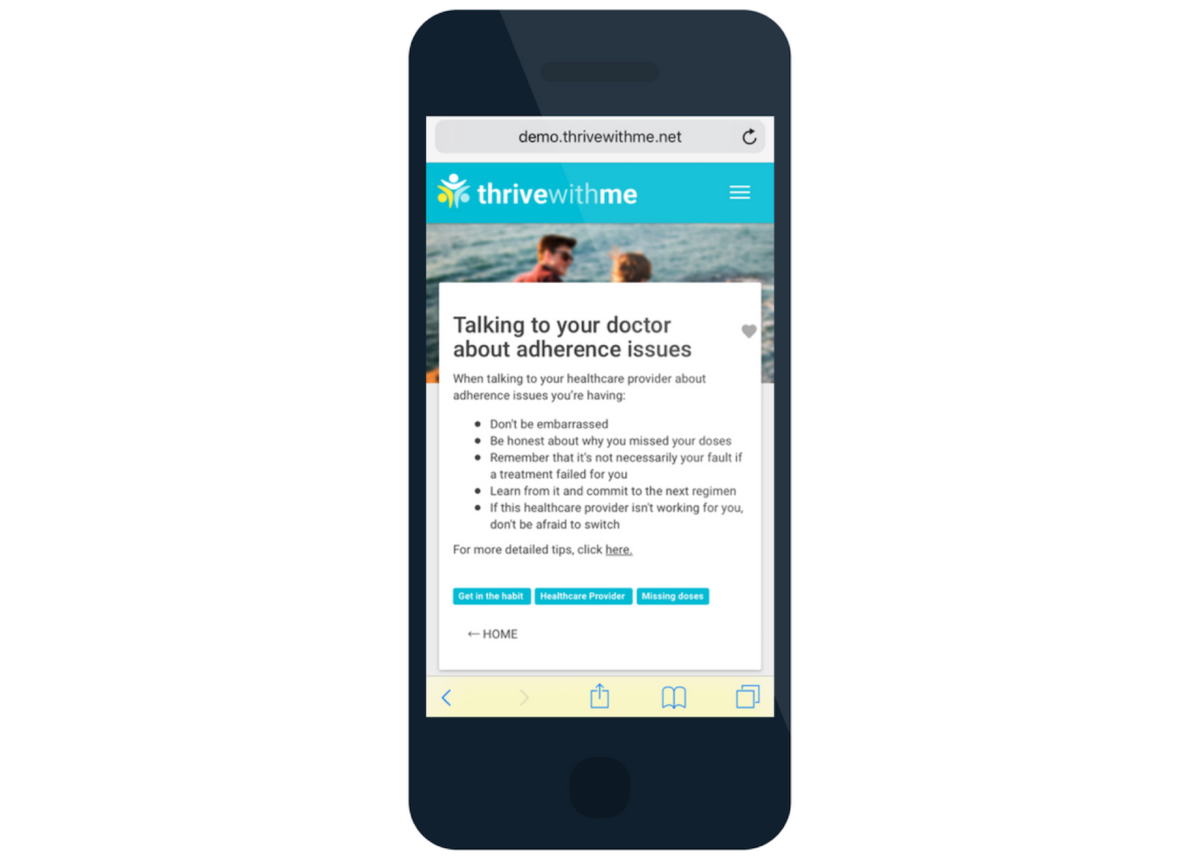
Thrive with Me “Thrive Tip”.

**Figure 5:**
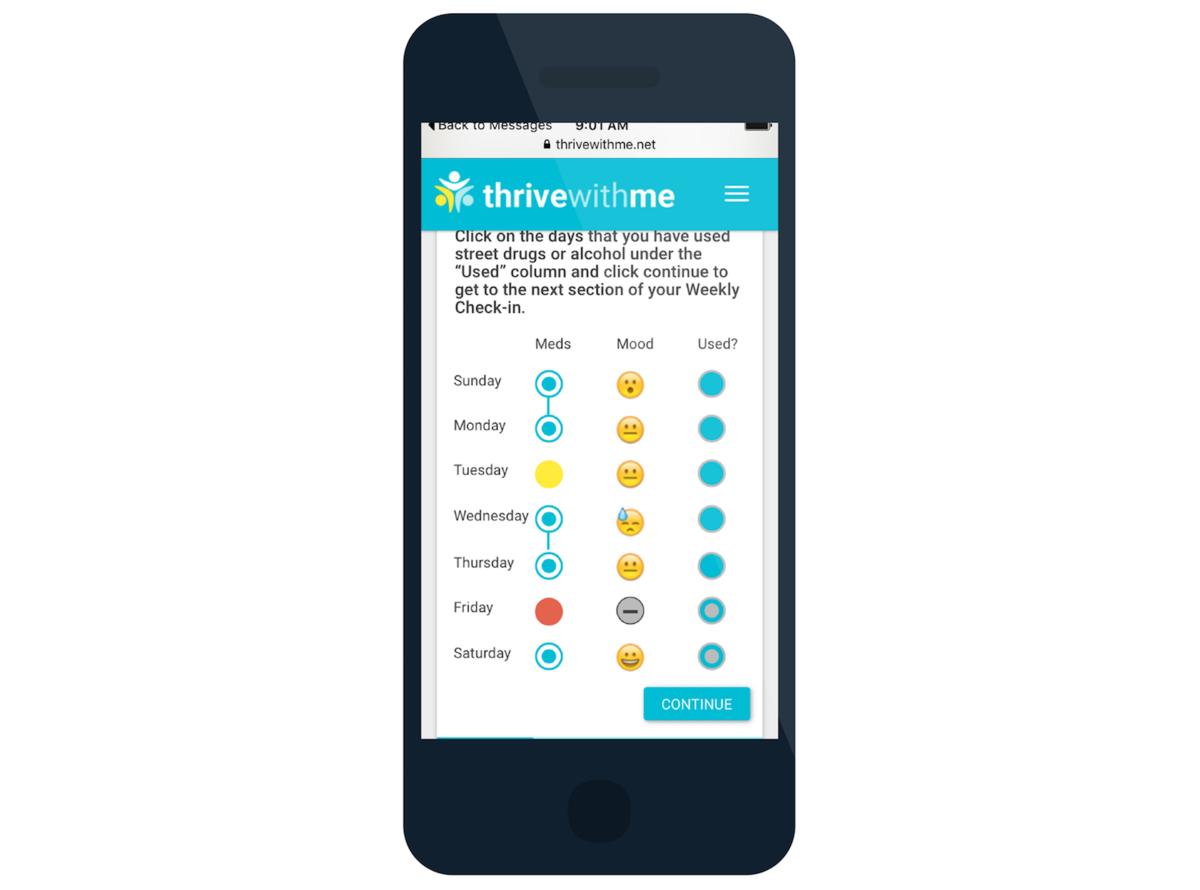
Thrive with Me adherence, mood, and substance use self-monitoring.

The active intervention period is 150 days (approximately 5 months). Men in the TWM experimental intervention arm have access to the full TWM website that has three primary components: (1) a private social networking feature (Figure 3); (2) tailored HIV and ART adherence information (Figure 4); and (3) medication reminders, self-monitoring, and reflection (Figure 5). Privacy on the TWM site was prioritized in several ways. First, TWM was designed to be a closed community, such that only participants could gain access to enter the site. Second, because of stigma that HIV-positive MSM commonly face and concerns that some participants may feel uncomfortable disclosing their identities online, we established community rules that men could not post pictures of themselves on the site. Men could choose from a number of avatars that gave them the opportunity to tailor their profile without disclosing their identity.

Using asynchronous peer-to-peer interaction, the TWM website was developed to be a safe space for supportive peer-to-peer interactions to promote healthy choices related to living with HIV and medication adherence (Figure 3). Men are encouraged to participate in the TWM social network as much as they are comfortable, similar to other social networking sites. TWM is moderated daily by research staff to identify any posts that appear to reflect self or other harm intentions or behavior, any hostile interactions, or misinformation that is not corrected by group members. TWM staff members post to the feed a few times a week to welcome new members to the site, remind participants of some features of the site, and to announce the winner of a weekly prize drawing for those who are very active on the site.

The TWM website presents HIV-related content to users every day in the form of Thrive Tips (Figure 4). Thrive Tips are content pieces (text, images, videos) that address barriers to medication adherence. There are 300 total Thrive Tips that are released 3 to 4 per day, each tied to an individual item in the IMB ART Adherence Questionnaire (IMB-AAQ; described below). Thrive Tips that reflect a participant’s unique ART adherence barrier are shown with a blue triangle in the corner of the tip to indicate to him that the information is particularly relevant to his needs. The longer participants are in the intervention, Thrive Tips are accumulated and stored in an online searchable library. Thrive Tips can be searched by category of information, content recommended for the specific user, content that has been favorited by the user, and tag words. Men retake the IMB-AAQ at the midpoint of the intervention, and all Thrive Tips are displayed again, although reflecting participant’s updated adherence barrier profile.

In addition, men receive daily short message service (SMS) reminders to take their medication at a time of their choosing. Men receive follow-up SMS messages asking them to report whether they took their dose that day (or not) and what their current mood is (Figure 5). Once a week, participants are reminded to complete their “weekly check-in,” where they are shown their responses to the daily SMS messages and are asked to report on which days in the prior week they used substances (which could include alcohol and drugs). To encourage reflection, men are then prompted to answer a multiple choice question about how their week was overall (ie, “Looking back at your week, how did you handle the tough things you had to do? 1 hot mess; 2 fell on my face; 3 don’t stop me now; 4 I will survive; 5 Sasha Fierce”) and are given a private text area to record anything from the week that they want to remember. The response options asking men to rate how their week was overall were intended to be engaging to the user, rather than to collect validated data on their perceptions of the quality of their week. Therefore, we did not assess beforehand whether HIV-positive MSM interpreted these items as ordinal. Participants may review past weeks of responses during the active intervention period.

**Figure 6:**
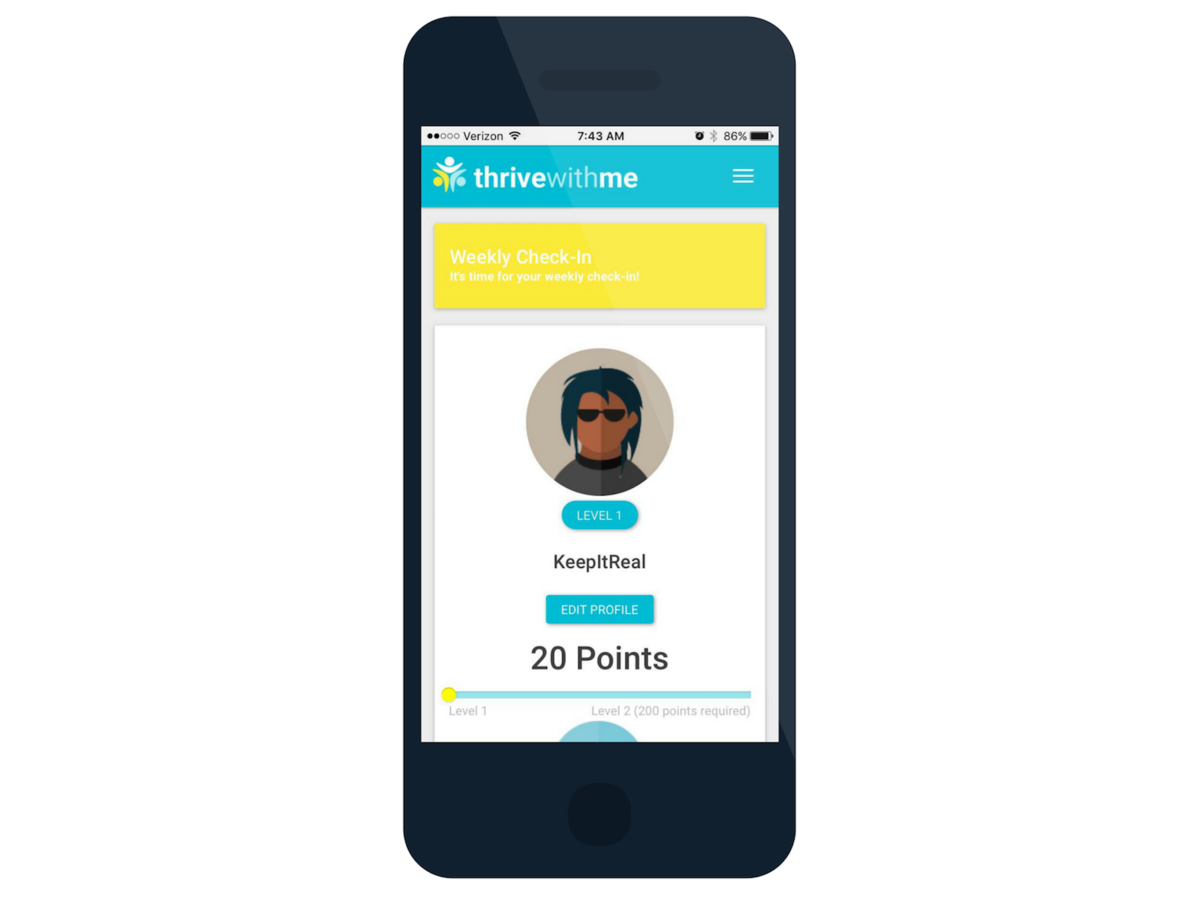
Thrive with Me profile and gamification.

**Figure 7:**
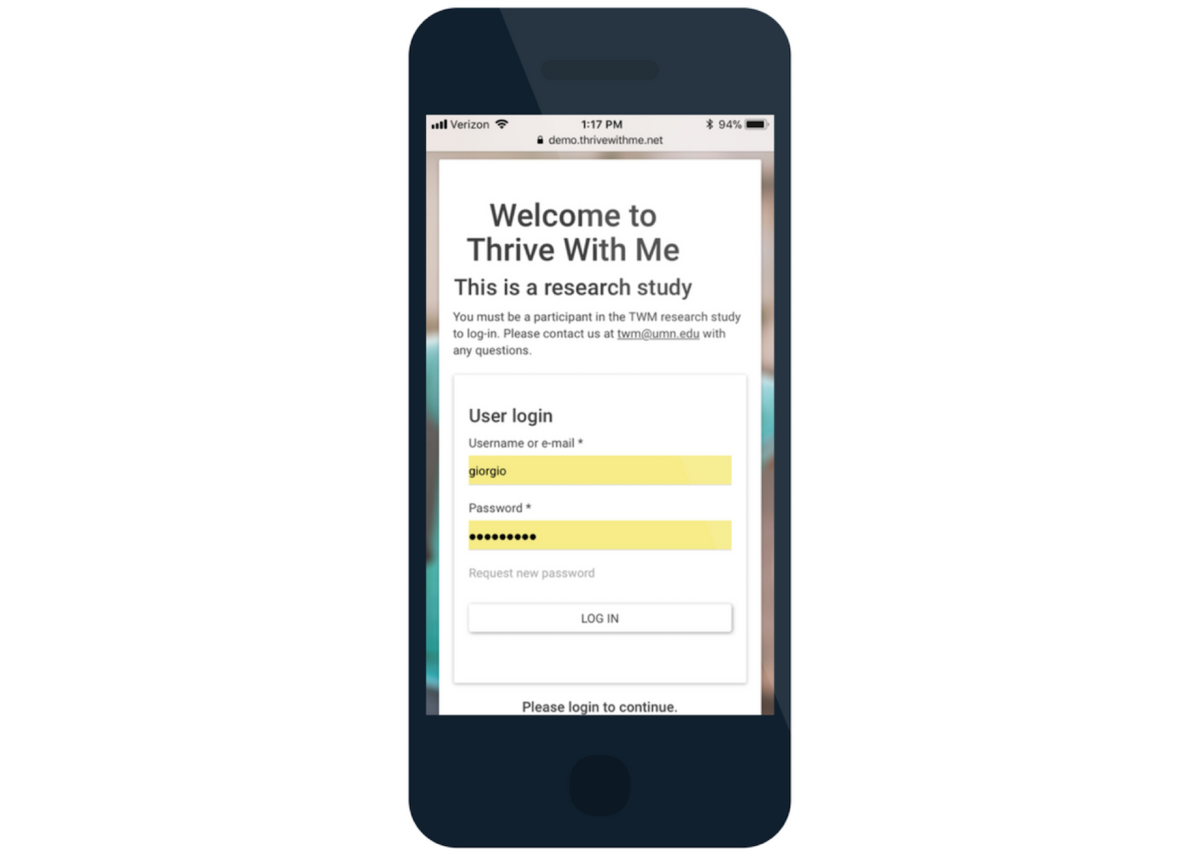
Thrive with Me log-in page.

In addition to these three primary components of the TWM intervention, other features of the site include (1) A profile page where men can update their profile and settings, gaming features (eg, points, achievements, and badges; Figure 6); (2) A weekly prize drawing of a US $25 online gift card for those who use the site 5 or more times in the past 10 days; (3) An “About us” page that describes the study and the study team; and (4) A “Getting started” page that provides instructions about how to use the TWM site. Access to the TWM site is given to participants who have the correct username and password and to study staff with the appropriate credentials (Figure 7).

#### Control Condition

Participants assigned to the control condition receive a weekly email with content similar to a newsletter for 21 consecutive weeks. Each email contains a link with information on a topic related to living with HIV and devoted to improving general well-being but not specifically about ART adherence. Sample topics of the information-only control content include HIV and longevity, HIV and parenting, and HIV and depression. Control content is developed in, and sent from, Qualtrics, to allow for the collection of data on which emails participants open. In addition, at the bottom of each informational page is the question “How would you rate this week’s content?” with a 5-star rating from the numeric anchors 1 to 5.

Both the TWM intervention and the control intervention weekly emails are viewable across multiple devices, including smartphone devices, tablet computers, and desktop and laptop computers.

### Participant Monitoring and Monitoring Adverse Events

#### Participant Monitoring

Participants will be informed during the consent process of the “group rules” regarding interactions with one another (eg, “Honesty is important, however, hostile or abusive language will not be tolerated and may be grounds for immediate removal from the study”). These “rules” will also be available with a link on the TWM site. The project coordinator will manually review each day posts that participants write to each other to flag hostile interactions and inaccurate information. Hostile interactions between participants will be handled by, first, reminding the participants in the interaction of the “group rules” regarding appropriate interactions. If the hostility continues, the offending participants will be given a warning that the continued hostility will result in withdrawal from the study if it continues. On the third offense, the offending participant will be withdrawn from the study. Text containing hostile exchanges will be removed from the study website and unavailable to view. In cases in which inaccurate information is found, project staff be guided by exerts on the team to post a comment that provides accurate information on the topic.

#### Monitoring Adverse Events

Study staff will identify adverse events (AEs) and serious adverse events (SAEs) that may occur during the study period. Such events will be immediately brought to the attention of the PI and the investigator team. AEs will be reported to the UMN Institutional Review Board (IRB; 1504S69721) within 5 working days. In the event that the UMN IRB requests follow-up reports regarding an AE, it is the responsibility of the study Pl to submit the report on or before the date requested by the IRB. SAEs will be reported to the UMN IRB and National Institute on Drug Abuse (NIDA) within 72 hours. Subsequent follow-up reports for a specific SAE will be submitted quarterly (ie, every 3 months) to NIDA. The follow-up report on any specific SAE will include correspondence from the IRB as to the determination of whether the SAE was related to the study. In the event that the UMN IRB requests follow-up reports regarding an SAE, it is the responsibility of the Pl to submit the report on or before the date requested by the IRB. The UMN IRB has the authority to suspend or terminate approval of any research at its site that has been associated with unexpected serious harm to participants. The conditions under which the study would be stopped include, but are not limited to, a direct causal link between the study conditions and the hospitalization, impairment, or death of any research participants. Finally, a data safety and monitoring board (DSMB) will be convened to determine safe and effective conduct and recommend conclusion of a trial if significant risks develop or the trial is unlikely to be concluded successfully. The board will consist of three experts in the field of HIV prevention and treatment intervention and will meet yearly.

### Preliminary Data

#### Community Advisory Board

Seven men who had participated in prior research at CHEST and indicated that they wanted to contribute to further research were given contact information for the TWM study site coordinator. Upon expressing interest in serving on a community advisory board (CAB), they were given a brief phone screening to ensure they were male-identified, at least 18 years of age, HIV-positive, currently on ART medications, lived in New York City, English speaking (as materials would all be in English), and had access to the internet. Four members of the CAB identified as African American or black, three identified as white, and two identified as Hispanic or Latino. CAB members were asked to complete three online surveys over the course of 2 months and were compensated US $50 per survey.

The first CAB survey gathered input on general user experience, particularly aesthetics, language, and usability. For aesthetics, we asked questions about a variety of color palettes and stock photography. Users were asked to rank favorites, given scales of adjective choices to describe aesthetics (ie, traditional vs modern or informal vs formal) and also were given free text options to include other feedback. Language questions were focused on wording of medication reminder SMS text messages, name choices for major features, what words we should use when talking about illicit drugs, and the naming of various badges and achievements. The second CAB survey was used to gather input for HIV-related content that was not directly tied to medication adherence. Suggestions regarding what topics to discuss mostly informed the development of content for the control arm. The third survey solicited feedback on a large assortment of avatar choices for user’s profiles, from abstract choices to different portrayals of people. It also included questions about a weekly self-monitoring component to assess clarity, usability, and overall tone of the language.

#### Beta Testing

A total of 16 men were recruited to beta test the TWM intervention. Beta testing included collecting both usability (ie, testing for navigation and technical errors) and acceptability (ie, gathering feedback about the intervention features, functions, and design) data. Inclusion criteria were male-identified, living in the NYC metropolitan area, HIV-positive, currently on ART medications, self-reported issues with medication adherence, English-speaking (as the website is in English), access to the internet, able to send and receive SMS messages, and available for follow-up interview.

Eligible men visited CHEST offices in person for two visits during beta testing. During the first visit, men completed a baseline CASI on which a majority of participants identified as non-Hispanic (81%, 13/16) and African American (75%, 12/16). The average age was 36 years. The majority of participants identified as homosexual or gay (94%, 15/16). Participants had been diagnosed with HIV for a mean of 11.6 years. All participants reported a history of lifetime drug use, whereas 13 (81%, 13/16) reported any drug use in the previous 30 days. With respect to ART adherence, participants on average missed 3.7 doses in the past 30 days.

After completing the baseline CASI, participants met individually with staff from CHEST for an orientation to the TWM website. CHEST personnel demonstrated all of the core components of the site from a desktop computer. Participants were also given the opportunity to log in on their mobile phones. Participants were given a “workbook” of tasks to complete on the TWM site over the next 2 weeks and note anything they liked, disliked, problems they encountered, or suggestions for improvement. Participants then scheduled their second visit for approximately 14 days later and were given US $50 for compensation. The first visit typically lasted 90 to 120 min in duration.

During the 2 weeks that men were using the website, research staff from the UMN and CHEST in New York City communicated daily about any technical challenges, participant questions, or other concerns. After 2 weeks of testing the website, participants returned to the offices of CHEST, and staff there facilitated a phone interview between the UMN study coordinator and the participant. The purpose of the call was to gain feedback from participants about problems they encountered as they used TWM, as well as their suggestions for improving the overall look and feel of the intervention. Users were logged into the TWM website during the telephone interview, and participants also had their notes from the workbook if they remembered to bring it with them to the second visit. Interviews typically lasted between 30 to 45 min, and participants were paid another US $50 to compensate for their time. All 16 participants completed both visits.

Beta testing feedback and how the feedback was incorporated (or not) into the final version of the TWM site is shown in Table 1. Most participants expressed positive opinions about the core features of the site; however, there were several areas in which participants noted opportunities for improvement. Feedback included both content and site design requests, as well as technical issues. Content feedback was incorporated into edits made by the UMN research team, whereas feature requests and technical issues were passed on to site developers. High priority feature requests included changing aspects of the site that were confusing or ungainly, increasing the customizability of users’ profiles, and altering the SMS reminder and check-in process. Low-priority feature requests were an orientation to using the site on a smartphone and more personalized options for the site.

Following a final revision of the TWM site based on feedback from beta testing, enrollment into the RCT (Figure 2) began in November 2016.

### Outcomes

The study outcomes below are organized as shown in Table 2. Measures are collected using an online survey tool (Qualtrics) at baseline and 5-month, 11-month, and 17-month postbaseline assessment time points. A table of measures administration is shown in Table 2.

**Table 1:** Participant feedback from beta testing of the Thrive with Me (TWM) site.

Topic	Likes	Dislikes	Participant recommendations	Action taken
SMS^a^ reminders	“The med reminders are amazing because they hit on the point, the exact time that I set it for, so it helps so much!...It helps me take my meds every day.”	“Sometimes it just says ‘bye’ at the end of the text message and that’s a little brief. Like maybe say something else or don’t say anything at all.”	New or different options to describe emotions, new valediction	Used more discrete language (multivitamin instead of dose) as default message; Mood responses updated; Changes to greeting messages and discontinued “goodbye” message
Thrive tips	“I think it’s amazing...that it gives you tips and like things I didn’t even know. I was like that’s awesome! I think they’re very helpful.”			Added Thrive Tips to the first week of the intervention specifically orienting participants to the intervention; Added Thrive Tips to the final 2 weeks of the intervention regarding finding social support after TWM, closure within the intervention and “graduating” from TWM
Mobile features	“On the computer it looked really nice and I liked the layout, like Facebook.”	“On the phone, I don’t know, it was more difficult to see everything, you had to scroll up and down and left and right and it was just hard to use.”	More mobile-friendly design and features, icon on phone's home screen	Updated layout of some Web pages; Added an icon for a phone’s home screen and provided participants instructions for setting the icon
Social features	“It was kind of helpful because I read how other people—how they expressed their emotions, how they felt, what they were going through…so it kind of helps you in your day by day so you don’t feel like you’re alone.”	“I wouldn’t use it for that. I mean, I don’t really want to engage with people who I don’t know.”	Larger maximum file size for uploads to post on the wall, notification when one's post is commented on or liked	Updated file size to accommodate larger images or graphic interchange formats; Built notification system to track comments and likes within the TWM site (no text, email, or banner notifications were added)
Profile	“Avatar choices are cute, they didn’t have faces, they are anonymous, but there should be more choices.”	“I don’t like the avatars at all. I’m half something and half something else with blue hair…It looks like a muppet.”	More diverse options for avatars, profile questions about HIV-related or life-related experiences	Increased avatar choices and added features requested by participants (glasses, afros, more feminine looking options, etc); Added “Talk to me about” feature with “check all that apply” responses for a variety of topics related to HIV and general well-being
Achievements	“They were cute, very cute…They’re uplifting, you know, you never know what someone is going through and some people are never told that they do anything right. The badges tell you ‘good job’.”	“Badges, achievements, I didn’t understand it. What are these points? Am I getting money at the end of the day? Why am I doing this?”	More clarity about what earns points and what badges mean	Added a “Getting Started with TWM” Web page to explain all components of the intervention; Updated in-person website orientation to include more details about achievements
Overall	“I mean we walk around with HIV, I have HIV positive friends, but a lot of times the only interaction I would have around HIV positive people would be around sex, so this kind of brings it around, where having that conversation brings it out of the sexual realm and maybe more into the supportive realm, for people who may not have that already.”	“For people newly diagnosed or early in their diagnosis this would be a very very useful tool…but for me personally it’s just not practical. I don’t need the things that it offers.”	N/A^b^	N/A

^a^SMS: short message service.

^b^N/A: not applicable.

**Table 2:** Schedule of measures administration. An “X” indicates that the measure was assessed at the corresponding time point. A “—” indicates that the measure was not assessed at the corresponding time point.

Variable		Measures	Assessment period			
			Baseline	5-month follow-up	11-month follow-up	17-month follow-up
**Primary outcome**						
	Viral load (VL)	Undetectable=VL<20 copies/mL	X	X	X	X
**Demographics and HIV history**						
	Age	Age in years	X	—	—	—
	Race or ethnicity	Race, ethnicity (Hispanic or Latino)	X	—	—	—
	Sexual orientation	Sexual orientation	X	X	X	X
	Education	Highest level of education completed	X	X	X	X
	Employment	Employment status, student status	X	X	X	X
	Income	Household income, number dependents on income	X	X	X	X
	Community	Population of residence	X	X	X	X
	Health insurance	Type of health care coverage	X	X	X	X
**Medical or clinical variables**						
	Self-reported HIV history	Year and month diagnosed with HIV, AIDS diagnosis	X	—	—	—
**Self-reported ART^a^ adherence**						
	Prescriptions	Number of prescribed medications, vitamins, and supplements	X	X	X	X
	Antiretroviral medications	Year started HIV medications, HIV medications currently taken, number of doses per day	X	X	X	X
	Adherence	Doses missed (past 4 days, 30 days), % adherence (past 30 days), % taken within 2 hours of scheduled dose (past 30 days), how good a job in taking HIV medications, challenges to adherence	X	X	X	X
	Prior adherence services	Previous adherence-related information or services received	X	X	X	X
**IMB^b^-ART Adherence Questionnaire**						
	Information	ART adherence information	X	X	X	X
	Motivation	ART adherence motivation	X	X	X	X
	Behavioral skills	ART adherence behavioral skills	X	X	X	X
**Engagement in HIV care**						
	Appointments	Last HIV care appointment, appointments scheduled and missed (past 6 and 12 months), upcoming appointment scheduled	—	—	X	X
	Engagement	Patient Activation Measure	—	—	X	X
**Substance use**						
	Urinalysis	Cocaine, methamphetamines, marijuana, and opiates	X	X	X	X
	Alcohol	Alcohol Use Disorders Identification Test	X	X	X	X
	Drug use	Drug use (lifetime) and frequency (past 30 days) of 14 illicit drugs (or other), ever sought help or treatment for alcohol or drugs	X	X	X	X
**Psychosocial risk factors**						
	Depression	Center for Epidemiological Studies-Depression 10-item scale measuring depressive symptoms in past week	X	X	X	X
	Stress	Perceived stress in past 30 days	X	X	X	X
	BSSS-4^c^	BSSS-4	X	X	X	X
	Life chaos	Measure of Life Chaos	X	X	X	X
	HIV stigma	HIV Stigma Scale (internalized, anticipated, enacted)	X	X	X	X
	Resiliency	HIV resilience items	X	X	X	X
**Relationship and sexual risk**						
	Relationship	Relationship status, relationship sexual agreement	X	X	X	X
	General sex	Number of sexual partners by gender (past 3 months)	X	X	X	X
	MSM^d^-specific sex	Male sexual contacts, frequency of anal sex and condomless anal sex; condomless anal sex by partner serostatus, partner pre-exposure prophylaxis use (past 3 months)	X	X	X	X
**Social support**						
	Social support	MOS^e^	X	X	X	X
	*TWM*^f^-specific social support	Emotional or information support from MOS, Social Support Questionnaire 6 within TWM	—	X	—	—
**Technology use**						
	Overall technology	Social media use, mobile phone use, service provider, device ownership, internet access	X	X	X	X
**TWM navigation and use**						
	TWM navigation	System Usability Scale, reasons for using TWM	—	X	—	—
	User engagement	Data captured while participants interact with TWM	X	X	X	X

^a^ART: antiretroviral therapy.

^b^IMB: information-motivation-behavioral skills.

^c^BSSS-4: Brief Sensation Seeking Scale-4.

^d^MSM: men who have sex with men.

^e^MOS: Medical Outcomes Study.

^f^TWM: Thrive with Me.

#### Primary Outcome

Blood draws will be taken to assess the effects of the *TWM* intervention on VL, one of the most objective and reliable indicators of ART adherence [2]. Blood will be drawn by a certified phlebotomist and analyzed by Quest Diagnostics. Plasma VL is considered undetectable at <20 copies/mL. Results of VL testing is provided to participants upon request.

#### Demographic and HIV History

Common demographic factors will be collected, including gender, age, race or ethnicity, sexual activity with men, income, educational attainment, employment status, and current health insurance. Self-reported HIV history factors include the year and month the participant first tested positive and whether or not they have ever received an AIDS diagnosis.

#### Self-Reported Antiretroviral Therapy Adherence

Participants are asked to report the medication they are currently taking, the total number of HIV medications taken, and the number of doses of HIV medication taken each day. Next, items adapted from Adult AIDS Clinical Trials Group [34] and from a more recent study by Wilson and colleagues assessing the validity of an ART adherence scale [35] are used to assess aspects of self-reported ART adherence. Items include the number of dosses missed in the past 4 days and in the past 30 days, how “good of a job” participants believed they are doing in taking their HIV medications in the way they are supposed to (from “very poor” to “excellent”), a visual analog scale to assess the percentage of HIV medications taken in the past 30 days and the percentage of HIV medications taken within 2 hours of the correct time in the past 30 days (both scales in 10 percent increments from 0 to 100), and what types of ART adherence support services they are currently receiving at their clinic.

To assess theoretically derived ART adherence strengths and barriers, IMB-AAQ [36] will be completed by participants, as it was in the TWM pilot trial [30,37], and by other research teams [18,30]*.* The IMB-AAQ is a 33-items measure that assesses adherence-related information (9 items), personal and social motivation (10 items), and behavioral skills (14 items) on a 1 to 5 Likert scale. Responses to these items will be used to tailor the presentation of the Thrive Tips to participants as described earlier.

#### Self-Reported Engagement in HIV Care

At the 11-month and 17-month assessment, participants will be asked to report the number of health care appointments for HIV they scheduled and missed in the past 6 months and past 12 months, how many months ago they most recently missed an appointment, and whether they have an HIV health care appointment scheduled in the next 6 months. The 13-item Patient Activation Measure [38] will be used to assess patient knowledge, skill, and confidence for self-management of their health care. The pattern of HIV care attendance and participants’ beliefs about the self-management of their health care will be used to characterize their engagement in HIV care.

#### Substance Use and Mental Health Variables

Substance use will be assessed in two ways. First, men will take urine screens at each assessment time point to assess for the following illicit substances: cocaine, methamphetamines, marijuana, opiates, and phencyclidine, using the Integrated E-Z Split Key Cup II-5 panel (Innovacon Laboratories). This test is capable of detecting drugs from 1 to 4 days after use, except for chronic marijuana use, which can be detected for up to 30 days [39]. Second, using items adapted from the TWM pilot study [30], lifetime and recent (past 6 months) use of the following illicit drugs will be assessed: marijuana, poppers (amyl nitrate), pain pills purchased on the street (oxycontin; percocet), powder cocaine, crack cocaine, amphetamine, methamphetamine, ketamine, ecstasy, heroin, cocaine and heroin mixed together, and hallucinogens. Participants also have the option to write in a drug not on the list. If a participant reported taking an illicit drug(s) in the past 6 months, he was asked to write in the number of times he used that drug(s) in the past 30 days. The Alcohol Use Disorders Identification Test (10-item alcohol abuse scale) is used to assess problematic alcohol use [40,41].

We will assess a broad range of mental health factors associated with ART adherence using widely used and validated standardized scales, including the Center for Epidemiological Studies-Depression (10-item depression scale) [42], the Perceived Stress Scale (14-items) [43], the 4-item Brief Sensation Seeking Scale [44], the 6-item Life Chaos Scale [45], and the HIV Stigma Scale [46]. In addition, 10 items from a HIV Resiliency Scale is used to assess the ability to cope with difficult life circumstances and positive beliefs about the self [47].

#### Relationship and Sexual Risk

We will assess each participant’s current relationship status (eg, “I am legally married” and “I am single and having sex with others”) and, if the participant reports being in a relationship, what agreement the participant has with his partner about sex outside the relationship (eg, “Both of us have sex with other separately” and “I have sex with others, but don’t know about my partner”) from items used in a prior study by members of the research team [48]. Next, we ask men to report the number of sexual partners they had in the past 3 months, who were male, female, and transgender (male and female separately). Finally, for participants’ male sex partners, we ask them to report the number of sexual contacts with men; the frequency of anal sex and anal sex without a condom; condomless anal sex acts with HIV-negative, HIV unknown, and HIV-positive partners; and the number of condomless sex acts with HIV-negative partners where the partner was on preexposure prophylaxis.

#### Social Support Variables

Two scales will be used to assess social support outside of and inside the TWM intervention. First, the *Medical Outcomes Study (MOS) Social Support Survey* (or *MOS*) is a validated 19-item instrument [49] that assesses the availability of four types of social support: emotional or informational, affectionate, tangible, and positive social interaction on a 1 (none of the time) to 5 (all of the time) scale. We will ask participants to complete the MOS at all time points to assess general social support. Additionally, we will use the 8-item emotional or informational support subscale (eg, “Someone to turn to for suggestions about how to deal with a personal problem”) to assess social support within the TWM intervention at the 5-month follow-up survey. Second, the *Social Support Questionnaire 6* [50] will be used to assess *who* provides support to whom in TWM and participants’ overall *satisfaction* with that support. Participants in the *TWM* intervention arm will be asked to indicate whether they received six types of specific support (eg, Whom can you really count on in the TWM study to help you feel more relaxed when you are under pressure or tense?). If the participant stated that he received a specific type of support, he is asked to report the username(s) of other participants in TWM who provided that type of support. Finally, men will be asked their overall satisfaction with the support they received during their time in TWM on a 6-point scale, from very dissatisfied (1) to very satisfied (6).

#### Technology Adoption and Use

Technology use questions and items assessing participants’ attitudes toward technology were taken from items developed by the Pew Research Center [51]. Men in TWM will report whether and how frequently they use social networking service (eg, Facebook and Instagram), what type of mobile phone they use, their service provider, the average number of SMS (ie, text) messages they send and receive in a month (with categorical response options), whether they own a desktop or laptop computer or a tablet computer, and where they access the internet. To assess how men in the study use their mobile phones, they are asked to state whether they have used their mobile phone for twelve activities (eg, looked up information about a health condition or reserve a taxi or car service).

#### Ease of Use of the Thrive With Me Intervention

Men randomized to the TWM intervention arm will be asked to rate ease-of-use of the TWM using the System Usability Scale (SUS) [52]. The SUS is a 10-item measure that asks men to rate on a 1 (strongly disagree) to 5 (strongly agree) scale how much they agree with statements about the ease with which they were able to navigate TWM (eg, “I found Thrive With Me unnecessarily complex” and “I found the various functions in Thrive with Me to be very well integrated”). Men in the control condition were not asked these items as they only received weekly emails.

#### User Engagement

Intervention use data will be collected during the active trial period to assess user engagement with the intervention. Intervention use data will include the following variables reflecting peer-to-peer interaction: (1) date of post, (2) original post content, (3) subject identity document (ID) of original post, (4) content of replies to the original post, and (5) subject ID of each reply. Additional user engagement variables collected are (1) Frequency of wall posts by subject ID, (2) Number of comments by subject ID, (3) Number of Thrive Tips viewed, (4) Number of recommended Thrive Tips viewed, (5) Number of responses to SMS medication reminders, (6) Number of mood responses reported, (7) Number of weekly check-ins completed, (8) Total number of active days, (9) Total points earned, and (10) Total badges earned (as well as which badges earned by participant ID).

### Statistical Analysis

Analyses will be performed using Stata or SE v13 or later. The study design is a 2 (condition: intervention vs control) X 4 (time: baseline, 5, 11, and 17 months) randomized trial, with condition a between-subjects effect and time a within-subjects effect. The two primary outcomes are dichotomous: undetectable VL (undetectable=VL <20 copies/mL vs detectable=VL >20 copies/mL) and adherence to ART (high adherence ≥90% adherence vs low adherence <90% adherence). Mixed-effect logistic regression models will be used to test hypotheses. These models will include main effects for condition and time and the condition × time interaction (a 3 degree of freedom comparison; the overall test of the hypothesis). We will also estimate a series of single degree-of-freedom comparisons (using planned contrasts) to estimate the effect of the intervention on adherence at the three postintervention follow-ups relative to baseline. We will primarily estimate effects as intention to treat. Efficacy of the *TWM* intervention will be indicated by a statistically discernable improvement in ART adherence and VL at 17 months.

To investigate whether there is greater benefit from the *TWM* intervention for drug-using participants compared with nondrug-using participants, we will use the approach described above with the two additional factors—self-reported current (past 5 months) drug use and the interaction with intervention (and time). In a similar manner, we will conduct exploratory analyses to identify other baseline participant risk groups for which the intervention is most beneficial. In addition, for participants in the *TWM* intervention, we will examine the effect of type and amount of intervention exposure (ie, which component usage are related to outcomes) on improvement in ART adherence.

### Incentives

Participants are paid US $50 in cash at the end of every in-person visit (baseline and 5-month, 11-month, 17-month assessments). Participants in the intervention group also are eligible to win a weekly prize drawing of a US $25 electronic gift card. Only active users who complete actions on the TWM site for 5 out of 10 days are eligible for the weekly prize drawing.

### Sample Size and Power Calculation

TWM will be tested in a two-arm RCT with a target N of 400 participants split equally between the intervention and control groups. Using variance and effect size estimates from the pilot study, power calculations determine that this sample size will allow for an 11 percentage point detectable difference in undetectable VL between intervention and control (eg, 71% undetectable vs 60% undetectable) at any of the three postintervention follow-up assessments relative to baseline (tested using planned contrasts described above). Detectable differences are similar for adherence and other secondary outcomes.

### Ethics Statement

All study procedures have been approved by the UMN IRB and Hunter College City University of New York Internal Review Board. One IRB does not cede to the other, and investigators from each institution report to their respective IRB.

A Certificate of Confidentiality has been obtained from the NIDA. Additionally, a DSMB has been established to provide regular oversight of research practices and activities to protect human subjects and the integrity of the data in the study. The DSMB comprised independent experts in the fields of HIV-positive populations, technology-based interventions, RCT methodology, and social support interventions. The study is registered on the national registry of clinical trials at clinicaltrials.gov; trial number NCT02704208.

## Results

The TWM RCT was launched in October 2016 and is ongoing. Baseline enrollment was completed in April 2018. Follow-up assessments will continue through August 2019.

## Discussion

Adherence to HIV medication continues to be problematic, with only 58% of HIV-positive persons aged 13 years or older in 37 states and the District of Columbia virally suppressed according to the latest surveillance report from the US CDC [6]. Suboptimal ART adherence is the result of a complex interplay between individual-, social-, societal-, and cultural-level factors, and primary reasons for missed doses can vary by sociodemographic dimensions [53-56]. Therefore, theoretically grounded behavioral interventions are needed to address these complex reasons for nonadherence among at-risk groups. However, in-person ART adherence interventions are often costly and require highly trained personnel to deliver intervention components. The TWM intervention leverages current mobile technologies (ie, a dynamic online website and SMS text messaging), as well as Web 2.0 technologies that allow users to generate and share content with each other [57], to impact ART adherence among drug-using and nondrug using MSM. Results of this efficacy trial would advance ART adherence science by providing evidence for this approach and, if effective, could inform subsequent interventions that also use either existing (eg, Facebook) or new social media platforms.

Technology-based behavioral interventions that use peer interactions to promote support have the potential to be highly engaging and use social motivation to create behavior change. However, there is limited knowledge on how to conduct these and other types of social media interventions. Important questions include (1) Is it more effective to use an existing social media platform (eg, Facebook) for intervention or develop a private virtual community? (2) What is the optimal way to monitor and manage activity within the community? (3) How do we engage users with each other in virtual communities? (4) What other features or components are needed to supplement the community feed to maximize benefit? And (5) When do we bring users into a virtual community (eg, immediately after being diagnosed with HIV or only after several months of adapting to their new diagnosis) and how frequently should users engage in them (ie, should they continuously be engaged or can users come in and out of the virtual community as needed) to be most effective? These and other questions about the use of virtual social support and other types of social media interventions will require a sustained research effort to answer. The lessons learned from the TWM study will begin to provide some answers to these questions to advance intervention science in this emerging area.

## Abbreviations

AE: adverse event
ART: antiretroviral therapy
CAB: community advisory board
CASI: computer-assisted survey instrument
CDC: Centers for Disease Control and Prevention
CHEST: Center for HIV Educational Studies and Training
DSMB: data safety and monitoring board
IMB: information-motivation-behavioral skills
IRB: institutional review board
MOS: Medical Outcomes Study
MSM: men who have sex with men
NIDA: National Institute on Drug Abuse
PI: principal investigator
PLWH: people living with HIV or AIDS
RCT: randomized controlled trial
SAE: serious adverse event
SMS: short message service
SUS: System Usability Scale
TWM: Thrive with Me
UMN: University of Minnesota
VL: viral load
